# Genetic and audiological determinants of hearing loss in high-risk neonates

**DOI:** 10.1016/j.bjorl.2024.101541

**Published:** 2025-01-03

**Authors:** Yanan Shi, Naiyao Zhang, Na Du, Tongxi Zheng, Ying Yu, Youjin Li

**Affiliations:** a Shanghai Jiao Tong University, School of Medicine, Hainan Branch of Shanghai Children’s Medical Center, Department of Otorhinolaryngology, Sanya, China; b Shanghai Jiao Tong University, School of Medicine, Hainan Branch of Shanghai Children’s Medical Center, Department of Neonatology, Sanya, China; c Shanghai Jiao Tong University, School of Medicine, Hainan Branch of Shanghai Children’s Medical Center, Department of Medical Genetics and Antenatal Diagnostic Center, Sanya, China; d Shanghai Jiao Tong University, School of Medicine, Shanghai Children’s Medical Center, Department of Otorhinolaryngology, Shanghai, China

**Keywords:** High-risk neonates, Hearing loss, Hearing Screening Test, Deafness gene screening

## Abstract

**Objective:**

We aimed to investigate the correlation between prevalent risk factors for high-risk neonates in neonatal intensive care unit and their hearing loss, and to examine the audiological features and genetic profiles associated with different deafness mutations in our tertiary referral center. This research seeks to deepen our understanding of the etiology behind congenital hearing loss.

**Methods:**

We conducted initial hearing screenings, including automated auditory brainstem response, distortion product otoacoustic emission, and acoustic immittance on 443 high-risk neonates within 7 days after birth and 42 days (if necessary) after birth. Neonates who failed initial screenings underwent further diagnostic tests at 3 months. The risk factors were analyzed retrospectively by Chi-Square test and stepwise logistic regression. Genetic analysis involved a deafness sequencing panel targeting 19 pathogenic variants across four genes (GJB2, GJB3, SLC26A4, and MT-RNR), applied to both the study cohort and a larger hearing screening cohort of 14863 neonates from our center and different medical centers in the same region.

**Results:**

Out of the 443 high-risk neonates, 222 failed their diagnostic hearing tests. Logistic regression identified preterm birth, neonatal hyperbilirubinemia and advanced maternal age (≧35 yr) as significant risk factors for hearing loss. Genetic screening of 33 neonates who failed the diagnostic tests revealed that 7 (21.21%) carried at least one pathogenic variant, with identified 1 homozygotes and 3 heterozygotes in the GJB2, 1 homozygotes and 1 heterozygotes in the SLC26A4 gene, and 1 homoplasmic variant in the MT-RNR (12SrRNA). In the larger hearing screening cohort, 497 (3.34%) were genetically positive for deafness mutations, among whom 29 had the diagnostic hearing tests and 7 eventually diagnosed with hearing loss. Of the rest 468 neonates who didn’t have the diagnostic tests, 445 (95.09%) passed the hearing screening tests.

**Conclusion:**

Preterm birth, neonatal hyperbilirubinemia and advanced maternal age are critical risk factors for hearing impairment in high-risk neonates. Mutations such as c.235delC in GJB2 and c.919-2A>G in SLC26A4 are the most common. Long-term follow-up of neonates carrying heterozygous variants, particularly in genes like GJB3, is necessary to understand their progression and hearing outcomes. This study highlights the importance of deafness gene screening in neonates to ensure accurate diagnosis and effective intervention.

**Level of evidence:**

Level 3.

## Highlights

• Preterm birth, NHB and AMA are potential risk factors of hearing impairment.
• Mutations such as c.235delC in GJB2 and c.919-2A>G in SLC26A4 are the most common.
• Deafness gene screening for neonates is important for diagnosis and intervention.

## Introduction

Hearing Loss (HL) has been the most common cause of disability.^1^ Around 0.5 to 5 per 1000 neonates and infants suffer from congenital or early childhood onset sensorineural deafness or severe-to-profound HL.^2^ The incidence of congenital HL in high-risk neonates was 10∼20 times higher than that of healthy newborn population.^2^ A long-term study of audiological follow-up among Neonatal Intensive Care Unit (NICU) graduates with sensorineural HL showed that nearly 40% of the patients showed a progressive hearing impairment.^3^ HL can have multiple deleterious effects on the new born most commonly being related to attainment of speech and language. It can also affect social, emotional and academic achievement of the child. Even mild or unilateral involvement may have detrimental effect on the development and on school performance of a young child.^4^

Early diagnosis of hearing impairment improves prognosis, hence screening programs have been widely and strongly advocated.^4^ The Joint Committee on Infant Hearing (JCIH)^5^ has recommended the 1-3-6 guidelines for the Early Detection and Intervention (EHDI) of HL. This program advises that hearing screening for neonates should be conducted by 1-month of age, definitive diagnostic testing completed by 3 months of age, and early intervention initiated by 6 months of age.

High-risk neonates in NICU suffer more various pathologies and risk factors than neonates in healthy nursery, which contribute to an increased incidence of HL.^6^ Previous studies demonstrated that the risk factors of neonatal hearing impairment include preterm birth, Low Birth Weight (LBW), Hyperbilirubinemia (NHB) with exchange transfusion, asphyxia, infection especially cytomegalovirus infection, ototoxic drugs, family history of early, progressive or delayed onset permanent childhood HL, NICU for more than 5 days, and craniofacial malformation.^7^, ^8^ We investigate into the risk factors for failure in the first-time screening test and diagnostic hearing test among high-risk neonates in NICU and found that failure in the first screening correlated with preterm birth, very LBW, revised Advanced Maternal Age (AMA), neonatal NHB and APGAR score less than 8 and that failure in the diagnostic tests correlated with preterm birth, very LBW, twins, AMA and revised AMA (≥ 40 yr).^9^, ^10^

There is increasing awareness of the importance of an etiologic diagnosis, and genetic testing with Next-Generation Sequencing (NGS) has the highest diagnostic yield for congenital/early-onset sensorineural hearing loss, one of the most common hereditary disorders.^11^ Identification of the genetic etiology is vital for early prevention and optimal treatment decisions. For instance, in the case of auditory neuropathy with mutations in the OTOF gene, patients are more likely to have their auditory nerve function preserved.^8^ If mitochondrial DNA variants are found, aminoglycosides should be avoided.^12^ Furthermore, deafness gene screening can help identify the causative genes, which will be helpful for accurate genetic counseling, prognosis, and the potential development of possible gene therapy strategies in the future.^12^, ^13^ We then further investigate the deafness gene mutation profiles of the high-risk neonates with hearing loss and then the audiological features of a larger cohort of neonates with different deafness gene mutation profiles from different centers in the same district in order to better understand the etiology of congenital hearing loss.

## Methods

This study was a retrospective cross-sectional study, in which we analyzed the association of common risk factors with the results of hearing tests (screening or diagnostic) in high-risk neonates in NICU. We also investigate the deafness gene mutation profiles and audiological features of a larger cohort of neonates from our tertiary referral center and other medical centers in the same district. All the data were collected within the framework of the newborn hearing screening program. The study protocol was approved by the Committee on Human Research of Hainan Hospital of Shanghai Children’s Medical Center Affiliated to Shanghai Jiaotong University School of Medicine (SYFYIRB(K)2024071).

### Subjects

A total of 443 high-risk neonates were enrolled from NICU of Hainan Hospital of Shanghai Children’s Medical Center from January, 2022 to November 2023. Of them, 222 failed to pass the diagnostic hearing tests, including 83 males (37.39%) and 139 females (62.61%), Among these 222 neonates, 95 underwent C-section (42.79%), with the gestational age at birth of 38.5 ± 2.12 weeks.

We also performed the deafness gene screening upon a larger cohort of 14863 neonates from our tertiary referral center and other hospitals in the same district.

### Hearing screening and diagnostic tests

**Fig. 1:**
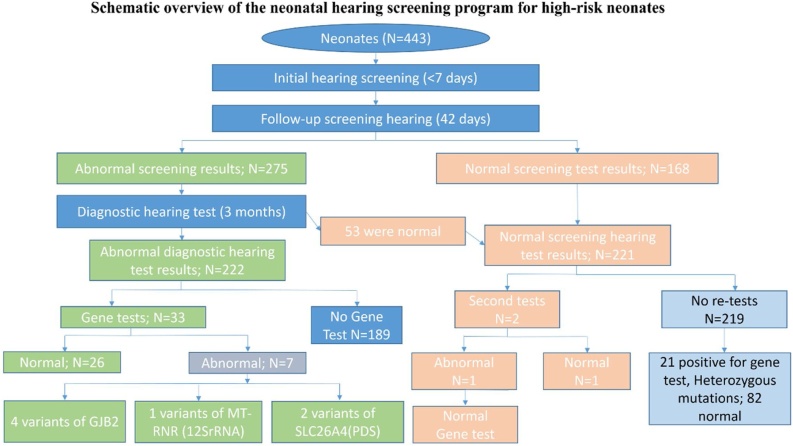
Schematic overview of the neonatal hearing screening program for high-risk neonates.

We performed Automated Auditory Brainstem Response (AABR), Distortion Product Otoacoustic Emission (DPOAE), and 1000 Hz probe tone Acoustic Immittance (AI) for the initial hearing screening test for all high-risk neonates within 7 days after birth. Infants who failed the AABR, OAE or AI test in one or both ears were classified as hearing screen failure, and AABR, DPOAE and AI were retested for them 42 days after birth. Comprehensive diagnostic hearing tests including Auditory Brainstem Response (ABR), DPOAE and AI were performed upon infants who failed in the retest or failed in the first test but didn’t have the retest 3 months after birth. All hearing tests were analyzed by a single audiologist blind to the medical records of the neonates. A schematic overview of the neonatal hearing screening program for infants is shown in Fig. 1.

### Deafness gene screening

A sequencing panel (BGI Genomics, Shenzhen, 518081, China) with 20 definite pathogenic variants (GJB2:c.176_191del16, c.235delC, c.235delG and c.299_300delAT; GJB3:c.538C>T and c.547G>A; SLC26A4: c.1174A>T, c.1226G>A, c.1229C>T, c.1975G>C, c.2027T>A, c.2168A>G, c.2162C>T, c.281C>T, c.589G>A, IVS15+5G>A (c.1707+5G>A) and IVS7-2A>G (c.919-2A>G); MT-RNR (12SrRNA): m.1095T>C, m.1494C>T and m.1555A>G) were selected for screening in this study.

Dried blood spots were collected via heel prick from neonates, and genomic DNA was extracted from these samples. The target regions (exons and splice site sequences of genes) in genomic DNA were captured using a set of oligonucleotide probes, followed by detection using high-throughput sequencing technology.

### Statistics analysis

The Pearson Chi-Square (χ^2^) test was used in comparisons between AABR and DPOAE results and in the correlation analysis between diagnostic hearing tests results and risk factors. The correlation between first hearing tests pass rates and factors were analyzed retrospectively by both univariate logistic regression and multivariate stepwise logistic regression analysis. All the analyses were performed with Stata 15.0 (Stata Corp, College Station, TX, USA), and *p* < 0.05 were considered statistically significant.

## Results

### Hearing screening and diagnostic evaluation of high-risk neonates

Among the cohort of 443 high-risk neonates, 275 (62.08%) didn’t pass the hearing screening tests in at least one ear and had to be referred, among whom the diagnostic yield of 128 (28.89%) AI, DPOAE and AABR was 128 (28.89%), 252 (56.88%) and 265 (59.82%), respectively. Three months after birth, 222 (50.11%) didn’t pass the diagnostic hearing tests, among whom the diagnostic yield of AI, DPOAE and ABR was 76 (17.16%), 202 (45.60%) and 215 (48.53%), respectively. Of the 215 neonates who failed the diagnostic ABR, the gold standard, 135 (62.79%) were diagnosed with mild hearing loss, including 43 (20%) moderate, 23 (10.70%) severe and 14 (6.51%) profounds. Therefore the total refer rate of the hearing diagnostic test was 48.53% (215/443).

### Deafness gene screening results of high-risk neonates

Thirty-three high-risk neonates underwent the deafness gene screening, and 7 (21.21%) patients were found to be positive. c.235delC variant of GJB2 were noted in 3 neonates, of whom one was homozygous with bilateral profound hearing loss. Heterozygous c.299_300delAT variant of GJB2 was positive in 1 neonate with bilateral mild hearing loss. Homoplasmic m.1095T>C variant of MT-RNR (12SrRNA) was noted in 1 neonate with left mild hearing loss. Homozygous c.2168A>G variant and heterozygous IVS7-2A>G (c.919-2A>G) of SLC26A4 were positive in 2 neonate, respectively (Table 1).

**Table 1:** Genetic variants in a deafness sequencing panel and hearing diagnostic outcomes in 7 neonates who failed hearing tests.

Gene Screening			Air ABR (dBnHL)		Hearing Tests
Gene	Mutations Alleles	Results	Right	Left	
GJB2	c.235delC	Homozygous	>100	>100	Bilateral profound
GJB2	c.299_300delAT	Heterozygous	40	40	Bilateral mild
GJB2	c.235delC	Heterozygous	35	20	Left normal, right mild
GJB2	c.235delC	Heterozygous	30	25	Left normal, right mild
SLC26A4 (PDS)	IVS7-2A>G (c.919-2A>G)	Heterozygous	25	35	Bilateral mild
SLC26A4 (PDS)	c.2168A>G	Homozygous	90	55	Left moderate, right severe
MT-RNR (12SrRNA)	m.1095T>C	Homoplasmic	30	35	Left mild, right normal

ABR, Auditory Brainstem Response.

### Correlation between diagnostic hearing evaluations and pregnancy/neonates related risk factors

**Fig. 2:**
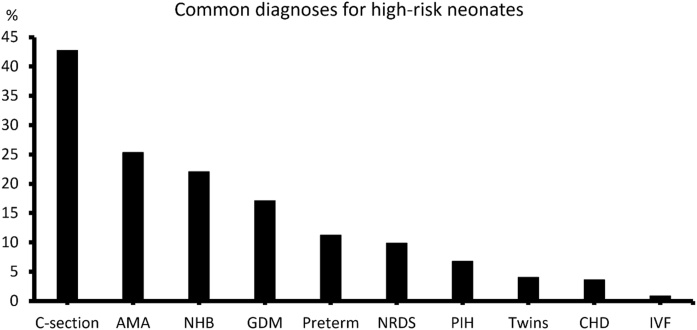
Common diagnoses for high-risk neonates. NHB, Neonatal Hyperbilirubinemia; LBW, Low Birth Weight; CHD, Congenital Heart Disease; AMA, Advanced Maternal Age; GDM, Gestational Diabetes; PIH, Pregnancy Induced Hypertension Syndrome; IVF, In Vitro Fertilization.

The neonate-related risk factors included preterm birth 25 (11.26%), Neonatal Hyperbilirubinemia (NHB) 49 (22.07%), APGAR score < 8 (22, 9.91%), Congenital Heart Disease (CHD) 8 (3.6%), twins 9 (4.05%), and In Vitro Fertilization (IVF) 2 (0.9%). The pregnancy-related risk factors included Advanced Maternal Age (AMA) 126 cases ≥ 35 year, 22.03% and 19 cases ≥ 40 year, 3.32%; Pregnancy-Induced Hypertension syndrome (PIH) 15 (6.76%); and Gestational Diabetes Mellitus (GDM) 38 (17.12%) (Fig. 2).

Univariate logistic regression analysis showed that none of the three tests were correlated with any risk factors. Stepwise logistic regression analysis indicated that the ABR failure rate of the first hearing screening was positively correlated with preterm birth (*p* *<* 0.05) and the failure rate of AI was positively correlated with NHB (*p* = 0.045) and AMA (*p* = 0.008) (Table 2).

**Table 2:** Correlation between diagnostic hearing evaluations and pregnancy/neonates related risk factors.

	Number (%)	Univariate Logistic Regression			Regression		
	n = 214	p (ABR)	p (DPOAE)	p (AI)	p (ABR)	p (DPOAE)	p (AI)
Sex (boy, %)	83 (38.79)	0.304	0.980	0.817	NA	NA	NA
Preterm birth	25 (11.26)	0.170	0.661	0.370	0.05*	NA	NA
NHB	49 (22.07)	0.457	0.832	0.815	NA	NA	0.045^a^
APGAR<8	22 (9.91)	0.888	NA	0.337	NA	NA	NA
AMA	48 (21.62)	0.319	NA	0.004	NA	NA	0.008^b^
CHD	8 (3.60)	NA	NA	0.412	NA	NA	NA
PIH	15 (6.76)	NA	0.149	0.405	NA	NA	NA
GDM	38 (17.12)	0.960	0.943	0.342	NA	NA	NA
Delivery	95 (42.79)	0.234	0.487	0.323	NA	NA	NA
IVF	2 (0.90)	NA	NA	0.936	NA	NA	NA
Twins	9 (4.05)	NA	0.960	0.485	NA	NA	NA

SW, Stepwise; NA, Not Applicable; ABR, Auditory Brainstem Response; DPOAE, Distortion Product Otoacoustic Emission; AI, Acoustic Immittance; NHB, Neonatal Hyperbilirubinemia; CHD, Congenital Heart Disease; IVF, In Vitro Fertilization; AMA, Advanced Maternal Age; PIH, Pregnancy-Induced Hypertension syndrome; GDM, Gestational Diabetes Mellitus.

a *p* < 0.05.

b *p* < 0.01.

### Deafness gene screening results of the hearing screening cohort

**Fig. 3:**
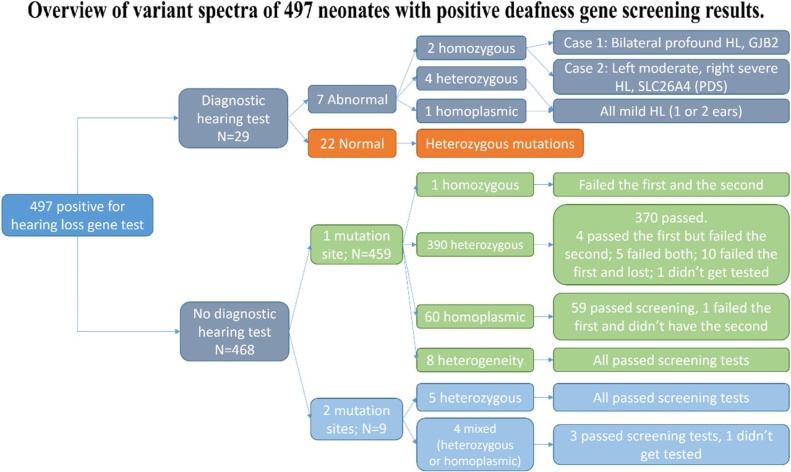
Overview of variant spectra of 497 neonates with positive deafness gene screening results.

Of all the 14863 neonates from different medical centers, 497 (3.34%, female/male = 214/283) were tested positive in at least 1 mutation allele, of whom 29 had the diagnostic hearing test, 466 had the screening test and 2 did not have any hearing test. Among all the 29 patients with diagnostic test results, 22 had normal hearing with one heterozygous variant, 5 had mild HL (4 heterozygous and 1 homoplasmic), 1 had bilateral profound HL with the homozygous variant of GJB2 (c.235delC) and 1 had left moderate/right severe HL with the homozygous variant of SLC26A4 (c.919-2A>G). Of all the 468 neonates without the diagnostic hearing test, 459 carried only one variant (1 homozygous, 390 heterozygous, 60 homoplasmic, and 8 heterogeneity), and 9 carried two variants in distinct genes (Fig. 3). Among them, 445 (445/468, 95.09%) passed the hearing screening tests. The detailed variant spectra were shown in Table 3.

**Table 3:** Positive gene screening tests in 497 neonates from different medical centers in the same region.

Gene	Mutation Alleles	Type	Number of Cases	Total number	Gene %
GJB2	c.176_191del16	Heterozygous	5	260	51.38 (95% CI 47.03‒55.74)
	c.235delC	Heterozygous	223		
		Homozygous	1		
	c.299_300delAT	Heterozygous	31		
SLC26A4 (PDS)	c.281C>T	Heterozygous	1	147	29.05 (95% CI 25.1‒33.01)
	c.589G>A	Heterozygous	1		
	IVS7-2A>G(c.919-2A>G)	Heterozygous	98		
		Homozygous	1		
	c.1174A>T	Heterozygous	5		
	c.1226G>A	Heterozygous	2		
	c.1229C>T	Heterozygous	11		
	IVS15+5G>A(c.1707+5G>A)	Heterozygous	5		
	c.1975G>C	Heterozygous	2		
	c.2027T>A	Heterozygous	2		
	c.2168A>G	Heterozygous	18		
		Homozygous	1		
MT-RNR1 (12SrRNA)	m.1095T>C	Homoplasmic	40	74	14.62 (95% CI 11.55‒17.7)
		Heterogeneity	1		
	m.1494C>T	Homoplasmic	2		
	m.1555A>G	Homoplasmic	25		
		Heterogeneity	6		
GJB3	c.538C>T	Heterozygous	17	25	4.94 (95% CI 3.05‒6.83)
	c.547G>A	Heterozygous	8		
Total			506	506	100

## Discussion

Early Hearing Detection and Intervention are advocated with birth hearing screening and intervention no later than 3∼6 months of age. This program has positively impacted outcomes for children suffering from deaf or hard of hearing and their families.^5^ In the present study, about 50% of the high-risk NICU neonates were diagnosed with different degree of hearing loss according to ABR test, although most of them were mild and might well recover with time.

Neonates may sometimes have retained amniotic fluid or infectious effusion in the middle ear, which can cause failure in ABR and OAE tests.^9^ Therefore, AI is needed to detect fluid and pressure in middle ears, thus increasing the specificity of SNHL diagnosis. The 1000 Hz probe tone AI is recommended before age 9 months for superior sensitivity and specificity than 226 Hz.^14^ The combination of AABR and OAE could also help screening auditory neuropathy. Therefore, as recommended by JCIH-2019,^5^ AABR, OAE and 1000 Hz probe tone AI should all be tested for neonatal hearing screening.

In the present study, preterm birth was an independent risk indicators positively associated with neonatal hearing loss, which is consistent with our previous studies.^9^ Preterm birth is often accompanied by LBW^15^ and these neonates are commonly exposed to other risk factors such as low APGAR score, intensive care treatment with mechanical ventilation, hypoxia, ototoxic drugs and NHB. It is been reported that the prevalence of neonatal hearing loss was reported to consistently increase with decreasing gestational age (1.2%∼7.5% from 31 to 24 weeks).^16^ In utero, hair cells are fairly mature from a morphologic standpoint sometime in the third trimester, but synaptic connections with the auditory nerve likely continue to mature after birth. It is estimated through ABR that the onset of hearing is about 27‒28 weeks, whereas peripheral maturation of the auditory system can generally be noted through Otoacoustic Emissions (OAEs) to be present at birth.^17^

With the rapid socioeconomic development, increasing respect for women’s rights and the “Late Marriage and Childbirth” propaganda since the opening-up policy in 1978, more women get married and then pregnant after 35 or even 40 years old. AMA was found to be negatively correlated with the pass rate of AI, which was consistent with the results in the previous studies of our hearing center.^9^, ^10^ As has been previously reported, women aged 45∼54, 40∼44, and 35∼39 years were at higher risk for a broad range of adverse outcomes such as C-section, preeclampsia and gestational diabetes.^18^ Actually, it has been well established in the literature that offspring exposed to unfavorable environmental conditions in utero, such as gestational diabetes, preeclampsia, and/or intrauterine growth restriction during the early stages of development are subject to long-term health consequences, including hearing loss.^19^ These potential risks should be taken into consideration seriously and examined carefully for women aged over 35 before and after pregnancy. In order to minimize the potential influence from other factors, we used stepwise logistic regression and found that the failure rate of AI was positively correlated with AMA (*p* = 0.008), which is consistence with our previous findings. Future studies should include more cases in our future research to better clarify if it could be regarded as a separate risk factor.

Bilirubin-induced auditory impairment primarily influences brainstem nuclei and the auditory nerve, leading to auditory neuropathy spectrum disorder.^20^ Auditory pathways are the sensitive parts of the nervous system to the toxic effects of bilirubin and total bilirubin level has the highest predictability for infant hearing status.^20^ In our study, we found that NHB was associated with lower pass rate of AI, but not with that of ABR or DPOAE, which was inconsistent with our previous reports.^9^ We speculated that most of them may suffer from temporary and mild NHB, but was timely and effectively treated, which significantly lowered the chance of hearing impairment. Further researches should follow-up more NHB neonates with different severities to better understand the influence of different levels of bilirubin on hearing and if possible, to clarify the underlying mechanisms through animal studies.

Principles and Guidelines for EHDI Programs recommended that every infant confirmed as deaf or hard of hearing, with or without middle ear dysfunction, should be referred by the medical home for specialty evaluations including otologic evaluation, genetics evaluation, et al.^5^ In our study, the detection rate of deafness genes by PCR hybridization screening was only 3.34%, which was comparable to previous genetic deafness testing results in Chinese newborns.^21^, ^22^, ^23^

Previous studies have identified 123 genes with variants associated with NSHL, among which GJB2 was a contributor to 50% of autosomal recessive Non-Syndromic Hearing Loss (NSHL) cases.^24^ GJB2 encodes the gap junction protein Connexin-26 (CX26) on chromosome 13q12.11, which variants and causes congenital sensorineural deafness.^25^ More than 100 variants in GJB2 have been identified, and the allele frequency displays obvious ethnic differences. The c.235delC variant was the most common variant, accounting for 86.15% (224/260, 1 homozygous), followed by heterozygous c.299_300del at 11.92 (31/260) and heterozygous c.176_191del16 at 1.92% (5/260), which was comparable to previous studies (Genetic screening of 15 hearing loss variants in 77,647 neonates with clinical follow-up).

SLC26A4 gene encoding pendrin is the second causative gene for NSHL. The neonates with SLC26A4 variation are at risk for Enlarged Vestibular Aqueduct (EVA) associated hearing loss, due to a chloride exchange imbalance in the inner ear from the embryonic to the postnatal period.^26^ The detection rate of SLC26A4 gene mutations in EVA patients in East Asia was approximately 80% and reached 97.9% in China.^27^ c.IVS8+1G>A is the most common mutation site in the SLC26A4 gene in Europe, while c.919-2A>G is the most common in the Chinese population.^28^ In this study, SLC26A4 contributed 34.92% of all mutations (147/497). IVS7-2A>G (c.919-2A>G) (99/147, 67.35%) and c.2168A>G (19/147, 12.93%) were the most 2 common mutation sites of SLC26A4, each of which 1 homozygous site was detected. The neonate with homozygous c.2168A>G mutation was diagnosed with left moderate, right severe HL and the one with homozygous IVS7-2A>G (c.919-2A>G) mutation failed both the first and the 42-day screening tests.

MT-RNR1 gene mutations are associated with HL because the mutations in this gene would create a binding site for aminoglycoside antibiotics, causing vestibular dysfunction and neuropathic HL on administration of these drugs.^29^ Genetic screening effectively reduced the incidence of drug-induced HL in recent decades, making these cases extremely rare. In our study, 17.58% (74/497 of mutation sites were found to be from MTRNR1, among which m.1095T>C was most common (41/74, 55.41%), followed by m.1555A>G (31/74, 41.89%) and m.1494C>T (2/74, 2.7%). Since mtDNA variants are maternally inherited, it may be reasonable to offer mtDNA variant screening to pregnant women, although lots of medical resources have to be used.

GJB3 causes delayed high-frequency phonological HL in an autosomal dominant manner; however, the pathogenesis of HL caused by these mutations remains unknown.^21^, ^30^ We noted 17 heterozygous mutation of c.538C>T and 8 heterozygous c.547G>A in the GJB3 gene, which is not very common. All of them have passed the hearing screening or diagnostic tests. The clinical manifestations of GJB3 related HL are difficult to detect and are frequently progressive, therefore, long-term follow-up of hearing changes is critical to this group of neonates.

The genotype–phenotype spectrum of hearing loss is broad. We reported 9 cases with 2 mutation sites, of whom the mixture of GJB2 and MT-RNR1 was noted in 2, followed by 3 mixture of GJB2 and SLC26A4, 2 mixture of GJB3 and SLC26A4, and 2 mixture of MT-RNR1 and SLC26A4. None of them was reported to have any risk factors for hearing. Although 8 of them passed the screening tests and 1 (GJB2 and MT-RNR1) was lost for follow-up, we should still remind parents for the possibility of late-onset hearing loss and attentions should be paid to hearing problems of children.

There are some limitations and need improvements in this study. First, only a small proportion of the neonates in NICU with abnormal diagnostic hearing tests results had the deafness gene screening, thus meriting further research to investigate the correlation of deafness gene expression and HL among high-risk neonates. Second, other risk factors such as assortative marriages, or chromosomal syndromes, should also be taken into consideration as risk factors. However, given the scope and design of our study, we focused only on high-risk neonates with acquired, non-syndromic causes commonly encountered in NICU settings. We did not systematically collect or analyze data on syndromic or chromosomal etiologies. Third, this study was limited by the sample size and follow-up duration of those neonates with abnormal diagnostic hearing results. Some delayed-onset HL (such as large vestibular aqueduct syndrome) or those with temporary auditory dysfunction or retarded central nervous system maturation might be missed or misdiagnosed, therefore leading to biases. A larger sample, longer-term follow-up studies are needed to further clarify the correlation of neonatal hearing loss and deafness gene expression.

## Conclusion

Preterm birth, neonatal hyperbilirubinemia and AMA are potential risk factors of hearing impairment. c.235delC of GJB2 and IVS7-2A>G (c.919-2A>G) of SLC26A4 (PDS) are the most common 2 mutation sites in this cohort. Long-term follow-up of neonates carrying heterozygous variants, particularly in genes like GJB3, is necessary to understand their progression and hearing outcomes. We therefore highlight the importance of deafness gene screening for neonates with hearing impairment for accurate diagnosis and optimal intervention.

## Funding

This study was funded by 10.13039/501100001809National Natural Science Foundation of China (82071015), 10.13039/501100003399Science and Technology Commission of Shanghai Municipality (21Y11900200), Shanghai Pudong New Area Health Commission (PW2022D-07), and Shanghai Key Laboratory of Clinical Molecular Diagnostics for Pediatrics (20dz2260900).

## Conflicts of interest

The authors declare no conflicts of interest.
